# Atovaquone-HSA nano-drugs enhance the efficacy of PD-1 blockade immunotherapy by alleviating hypoxic tumor microenvironment

**DOI:** 10.1186/s12951-021-01034-9

**Published:** 2021-10-02

**Authors:** Simeng Wang, Xinrui Zhou, Zekun Zeng, Mengjun Sui, Lihong Chen, Chao Feng, Chen Huang, Qi Yang, Meiju Ji, Peng Hou

**Affiliations:** 1 Key Laboratory for Tumor Precision Medicine of Shaanxi Province, The First Affiliated Hospital of Xi’an Jiaotong University, Xi’an, 710061 People’s Republic of China; 2 Department of Endocrinology, The First Affiliated Hospital of Xi’an Jiaotong University, Xi’an, 710061 People’s Republic of China; 3 International Medical Center, The First Affiliated Hospital of Xi’an Jiaotong University, Xi’an, 710061 People’s Republic of China; 4 Institute of Genetics and Developmental Biology, School of Basic Medical Sciences, Xi’an Jiaotong University Health Science Center, Xi’an, 710061 People’s Republic of China; 5 Center for Translational Medicine, The First Affiliated Hospital of Xi’an Jiaotong University, Xi’an, 710061 People’s Republic of China

**Keywords:** Hypoxic tumor microenvironment, Atovaquone, Anti-PD-1 therapy, Nano-drugs, Tumor targeting

## Abstract

**Background:**

Hypoxia is inherent character of most solid malignancies, leading to the failure of chemotherapy, radiotherapy and immunotherapy. Atovaquone, an anti-malaria drug, can alleviate tumor hypoxia by inhibiting mitochondrial complex III activity. The present study exploits atovaquone/albumin nanoparticles to improve bioavailability and tumor targeting of atovaquone, enhancing the efficacy of anti-PD-1 therapy by normalizing tumor hypoxia.

**Methods:**

We prepared atovaquone-loaded human serum albumin (HSA) nanoparticles stabilized by intramolecular disulfide bonds, termed HSA-ATO NPs. The average size and zeta potential of HSA-ATO NPs were measured by particle size analyzer. The morphology of HSA-ATO NPs was characterized by transmission electron microscope (TEM). The bioavailability and safety of HSA-ATO NPs were assessed by animal experiments. Flow cytometry and ELISA assays were used to evaluate tumor immune microenvironment.

**Results:**

Our data first verified that atovaquone effectively alleviated tumor hypoxia by inhibiting mitochondrial activity both in vitro and in vivo, and successfully encapsulated atovaquone in vesicle with albumin, forming HSA-ATO NPs of approximately 164 nm in diameter. We then demonstrated that the HSA-ATO NPs possessed excellent bioavailability, tumor targeting and a highly favorable biosafety profile. When combined with anti-PD-1 antibody, we observed that HSA-ATO NPs strongly enhanced the response of mice bearing tumor xenografts to immunotherapy. Mechanistically, HSA-ATO NPs promoted intratumoral CD8^+^ T cell recruitment by alleviating tumor hypoxia microenvironment, thereby enhancing the efficacy of anti-PD-1 immunotherapy.

**Conclusions:**

Our data provide strong evidences showing that HSA-ATO NPs can serve as safe and effective nano-drugs to enhance cancer immunotherapy by alleviating hypoxic tumor microenvironment.

**Graphic abstract:**

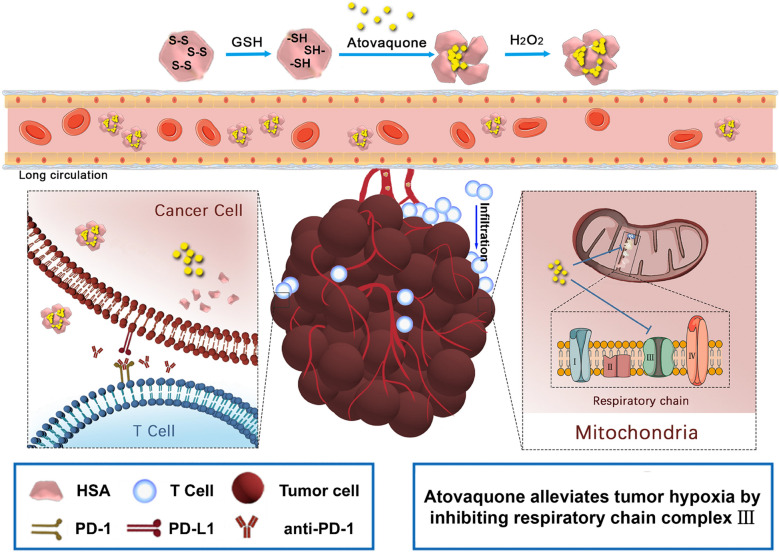

**Supplementary Information:**

The online version contains supplementary material available at 10.1186/s12951-021-01034-9.

## Introduction

Over the last decades, malignant cancer has posed a serious threat to public health and its incidence continues to be high [1, 2]. Up to date, immunotherapy has demonstrated to be a reliable therapeutic approach for most cancers [2–5]. One of the major advantages in cancer immunotherapy involves the antibody-mediated blockade of co-inhibitory “checkpoint” molecules, including cytotoxic T lymphocyte-associated protein 4 (CTLA4) and programmed death-1 (PD-1) [2, 4]. Blocking PD-1/programmed death ligand 1 (PD-L1) signaling has been proved to have durable antitumor immunity and remarkable clinical responses [6, 7]. Varieties of antibodies against the immune checkpoint proteins, PD-1 and PD-L1, have been widely used for cancer therapy [7]. Although some patients responded to immunotherapy, the majority of patients failed to gain ideal benefit, especially the patients with solid tumors [4, 8, 9].

In contrast to normal tissues, solid tumors have a more complex and harsher microenvironment [10–13]. Hypoxia is a hallmark of solid tumor physiology, as the proliferation of tumor cells results in deprivation of oxygen due to insufficient blood flow from abnormal tumor microvasculature [12, 14, 15]. Tumor hypoxia represents a great challenge in cancer therapy [12, 14, 16]. Intratumoral hypoxia increases tumor aggressing, chemo-resistance and radio-resistance [12, 13]. In recent years, many studies have reached a consensus that hypoxia could limit the efficacy of immunotherapy [17–19]. Meanwhile, there is evidence showing that normalizing tumor hypoxia microenvironment has a robust immune response [19, 20]. Minimizing mitochondrial oxygen consumption is considered to be one of the most effective approaches to eliminate hypoxia [20, 21]. Atovaquone, a small molecule drug, was approved by the Food and Drug Administration (FDA) to treat malaria and other diseases [22]. Previous studies indicated that atovaquone could decrease the oxygen consumption of solid tumors by inhibiting the mitochondrial complex III activity, eventually alleviating tumor hypoxia [22].

Due to the solubility of atovaquone in water is extremely low, oral preparation remains the most common dosage form for drug delivery [22, 23]. However, its low bioavailability greatly limits its clinical application. Given that albumin is a biocompatible and safe carrier, and most clinically approved nano-drugs are formulated in albumin [24, 25], thus albumin-based delivery system is a good choice to overcome solubility problem of atovaquone. Human serum albumin (HSA) is typically used for drug delivery due to its globular extremely stable structure, which be formed by internal 17 pairs of disulfide bonds [26]. During the process of disulfide bond disconnecting and reconnecting, atovaquone can be enwrapped in albumin, forming a nanoparticle. This approach will improve the bioavailability of the drugs and provide a tumor-targeting effect. Nanoparticles (NPs) are particularly attractive carriers for anticancer drugs because of their efficient cellular internalization and “enhanced permeability and retention (EPR)” properties [27, 28]. Besides, nano-albumin can be enriched in tumor sites by targeting to glycoprotein 60 (gp60) [24, 25].

In this study, we fabricated stable HSA nanoparticles loaded with atovaquone with a redox-responsive character, termed HSA-ATO NPs, and demonstrated that HSA-ATO NPs had excellent tumor-targeting property, and enhanced the efficacy of anti-PD-1 therapy by alleviating tumor hypoxia.

## Materials and methods

### Synthesis and preparation of HSA-ATO NPs

To cleave disulfide bonds of HSA to free sulfhydryl groups, HSA was added to deionized water containing 50 mmol/L GSH (glutathione) for 4–6 h at 37 °C. One mL of DMSO containing 10 mg of atovaquone was then added dropwise into 10 mL the above solution with slow stirring speed for 15 min. Next, 100 µL H_2_O_2_ (30% aqueous) was continuously added into the suspension to recover intermolecular disulfide bonds. Finally, the suspension was purified by dialysis (MWCO 10 kDa) followed by a freeze-drying to remove free atovaquone and DMSO.

### Physicochemical characteristics of HSA-ATO NPs

The average size and zeta potential of HSA-ATO NPs was measured by dynamic light scattering (DLS, Zetasizer NanoZS, Malvern, Worcestershire, UK). The morphology of HSA-ATO NPs was characterized by transmission electron microscope (TEM) (JEOL JEM-1200EX, Tokyo, Japan). To determine the drug loading and entrapment efficiency (EE), HSA-ATO NPs was first treated with tyrisin 1:2 (v/v) at 37 °C for 2 h, and then diluted in 2 mL of DMSO and sonicated for another 30 min to completely extract atovaquone. The atovaquone amount was determined on a high performance liquid chromatography (HPLC) system. Briefly, HPLC analyses were performed at 40 °C on a Waters XBridge C18 column (4.6 × 150 mm, 3.5 μm) running a 45 min, 5–95% linear gradient of acetonitrile in water containing 0.1% TFA at a flow rate of 1 mL/min.

### Cell culture

Murine colon adenocarcinoma cell line MC38 was obtained from the American Type Culture Collection (ATCC; Manassas, VA, USA), and routinely cultured in Dulbecco’s Modified Eagle Medium (DMEM) (Gibco, Grand Island, NY) supplemented with 10% fetal bovine serum (FBS).

### In vitro redox-responsive behaviors of HSA-ATO NPs

The ATO release experiment of HSA-ATO NPs was determined by dialysis. Briefly, 1 mL of purified HSA-ATO NPs (0.5 mg/mL ATO) solution was sealed into a dialysis bag (MWCO 100 kDa), and this bag was then immersed into 20 mL of PBS buffer (pH 7.4) containing 0.1% (v/v) Tween 80 with or without 10 mmol/L GSH. To maintain the silk condition, the solution was shaken at 100 rpm at 37 °C. An aliquot of release solution (200 µL) at 0, 2, 6, 12, 24 and 48 h was then withdrawn for HPLC assay.

### Seahorse assay

Using a Seahorse XFe96 Bioanalyzer (Agilent, Agilent Technologies, USA), MC38 cells (15,000/well) were plated on Seahorse culture plates in Seahorse required medium. Basal oxygen consumption rates (OCR) were taken for 20 min. Cells were then stimulated with 2 mmol/L oligomycin, 0.5 mmol/L FCCP and 100 mmol/L rotenone/antimycin A to obtain maximal respiratory and control values.

### Evaluation of tumor hypoxia microenvironment

At 90 min before tumors were surgically excised, pimonidazole hydrochloride (Hypoxyprobe™ RedAPC Kit, Hypoxyprobe, USA) was intravenously injected into mice at a dose of 50 mg/kg. The paraffin-embedded tissue slides were prepared, and then stained with anti-pimonidazole antibody-APC (Hypoxyprobe) and Hoechst 33342 (ThermoFisher, Beijing, China) after dewaxing and rehydrating. Finally, the sections were imaged with a confocal microscope (Leica, Leica Microsystems).

### Animal studies

The NOD/SCID and BALB/c nude mice (Beijing Vital River Laboratory Animal Technology Co., Ltd. Beijing, China) were used to establish patient-derived xenograft (PDX) model. In brief, tumor tissues were obtained from primary colon cancer tissue after surgical resection, and stored on iced DMEM supplemented with 10% FBS and antibiotics. Next, these tissues were immediately cut into 1–3 mm^3^ pieces, and subcutaneously implanted into the flanks of NOD/SCID mice. When tumor volume reached 1–2 cm^3^, xenograft tumors were harvested and cut into 1–3 mm^3^ pieces, and subcutaneously implanted into the BALB/c nude mice. When xenograft tumors grew to 100 mm^3^, (1) PBS, (2) HSA, (3) 6 mg/kg atovaquone, (4) 60 mg/kg atovaquone and (5) HSA-ATO NPs were intraperitoneally injected for 7 days. Tumor weight and volume were measured before xenograft tumors were harvested for further analysis.

The xenograft tumor model was similarly established as described previously [29]. When the average tumor volume reached ≈ 100 mm^3^, the mice bearing tumor xenografts were then randomly divided into different groups (nine mice per group), and the treatment was begun. HSA (60 mg/kg), HSA-ATO NPs (60 mg/kg, Atovaquone ≈ 6 mg/kg), atovaquone (6 mg/kg) and anti-PD-1 (100 µg each time) were administered at respective dose, with PBS as a negative control. Tumor volume was calculated, and immunohistochemical staining was then performed as described previously [29]. All of the experimental procedures were performed according to Institution Guidelines and approved by Laboratory Animal Center of Xi’an Jiaotong University.

### In vivo biodistribution analysis

At 6 h after treatment with a single dose of HSA-ATO NPs or atovaquone, the mice were sacrificed and the main organs and tumors were then isolated. Next, about 200 mg tissues were ground and homogenized followed by a 12 h DMSO extraction, and subjected to HPLC analysis.

### Biosafety evaluation of HSA-ATO NPs

The C57BL/6J xenograft tumor model was used to evaluate the biosafety of HSA-ATO NPs. Tumors and major organs (including liver and kidney) were collected from mice with different treatments for hematoxylin and eosin (H&E) assay. All of the images were obtained under an Olympus DP71 microscope. The examinations of liver and kidney function, including the levels of alanine transaminase (ALT), aspartate aminotransferase (AST), serum creatinine (CRE) and blood urea nitrogen (BUN), were performed as described previously [29].

### Flow cytometry analysis

After sacrificing tumor-bearing mice, MC38 tumors were dissected, cut into pieces and then incubated with DMEM media containing 5 mg/mL collagenase and 1 U/mL DNase I for 60 min at 37 °C. The homogenates were washed with PBS and passed through a 70 μm nylon mesh to acquire single-cell suspensions. For CD4+ or CD8+ T lymphocyte analysis, the cells were stained with fluorescence-labeled anti-CD45 (PE anti-mouse CD45 Antibody, Cat. #147711), anti-CD3 (APC anti-mouse CD3 Antibody, Cat. #100236) and anti-CD4 (FITC anti-mouse CD4 Antibody, Cat. #100405) or anti-CD8 (FITC anti-mouse CD8 Antibody, Cat. #100705). All antibodies were diluted according to the instructions and incubated with the cells for 30 min at room temperature. The cells were then analyzed by the BD FACSCalibur flow cytometer (BD Biosciences).

### Enzyme-linked immunosorbent assay (ELISA)

The Granzyme and IFN-γ content in tumor sites from mice were examined by the Mouse ELISA Kit (Jianglai Bio, Shanghai, China) according to the manufacturer’s protocol. The values were normalized by tissue weight.

### Statistical analysis

All statistical analyses were conducted using the SPSS statistical package (16.0, SPSS Inc. Chicago, IL). Unpaired student’s *t* test was used to compare the means of two groups of data. All values were expressed as the mean ± standard deviation (SD). All values with *P* < 0.05 were considered significantly.

## Results

### Atovaquone effectively alleviates tumor hypoxia by inhibiting mitochondrial activity of tumor cells

**Fig. 1:**
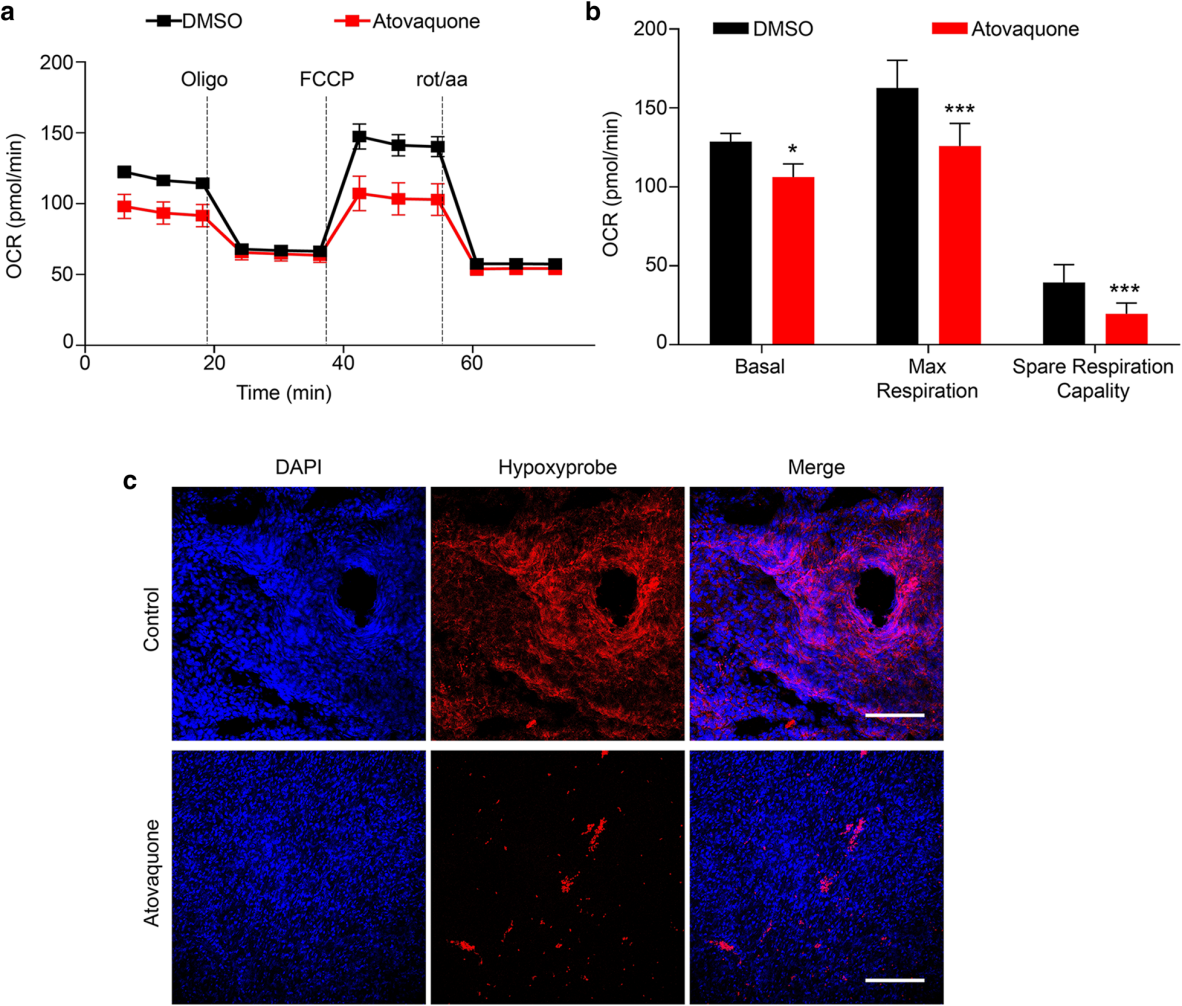
Alleviating tumor hypoxia by atovaquone via inhibiting mitochondrial complex activity of tumor cells. **a**, **b** OCR trace of MC38 cells (15,000 cells/well) interrogated for mitochondrial activity in the Seahorse instrument. **c** APC-Hypoxia probe staining of tumor sections from mice bearing MC38 tumors receiving PBS or atovaquone treatment for 7 days. Scale bar: 200 μm. Data are presented as mean ± SD. *, *P* < 0.05; **, *P* < 0.01; ***, *P* < 0.001

To validate the inhibitory effect of atovaquone on mitochondrial activity of tumor cells, we assessed tumor cells’ ability by Seahorse Bioanalyzer by measuring oxidative phosphorylation (OXPHOS), such as oxygen consumption rates (OCR). Before measuring OCR, a cytotoxic experiment was performed to define proper concentrations for subsequent experiments. The results showed that no more than 10 µM atovaquone almost did not affect the proliferation of MC38 cells (Additional file 1: Figure S1a), while 50 µM atovaquone showed pronounced cytotoxicity (Additional file 1: Figure S1b). Thus, we treated MC38 cells with 1 µM atovaquone for 48 h. Next, we sequentially injected oligomycin, phenylhydrazone (FCCP), rotenone and antimycin A to measure basal respiration, maximal respiration and spare respiratory capacity. The results showed that atovaquone clearly suppressed basal respiration, maximal respiration and spare respiratory capacity of MC38 cells compared to the control (Fig. 1a, b), indicating that atovaquone effectively inhibits OXPHOS process of MC38 cells.

To further determine whether atovaquone can alleviate tumor hypoxia, we first established a MC38 xenograft tumor model. When xenograft tumors reaching 100 mm^3^, each mouse was administered intragastrically with a single dose of 60 mg/kg (approximate 1.2 mg each mouse) atovaquone or sterile water once a day for 7 days. With the help of hypoxia-probes, atovaquone treatment clearly decreased overall tumor hypoxia (Fig. 1c). Taken together, our data indicate that atovaquone alleviates tumor hypoxia by inhibiting mitochondrial activity of tumor cells.

### Preparation and characterization of HSA-ATO NPs

**Fig. 2:**
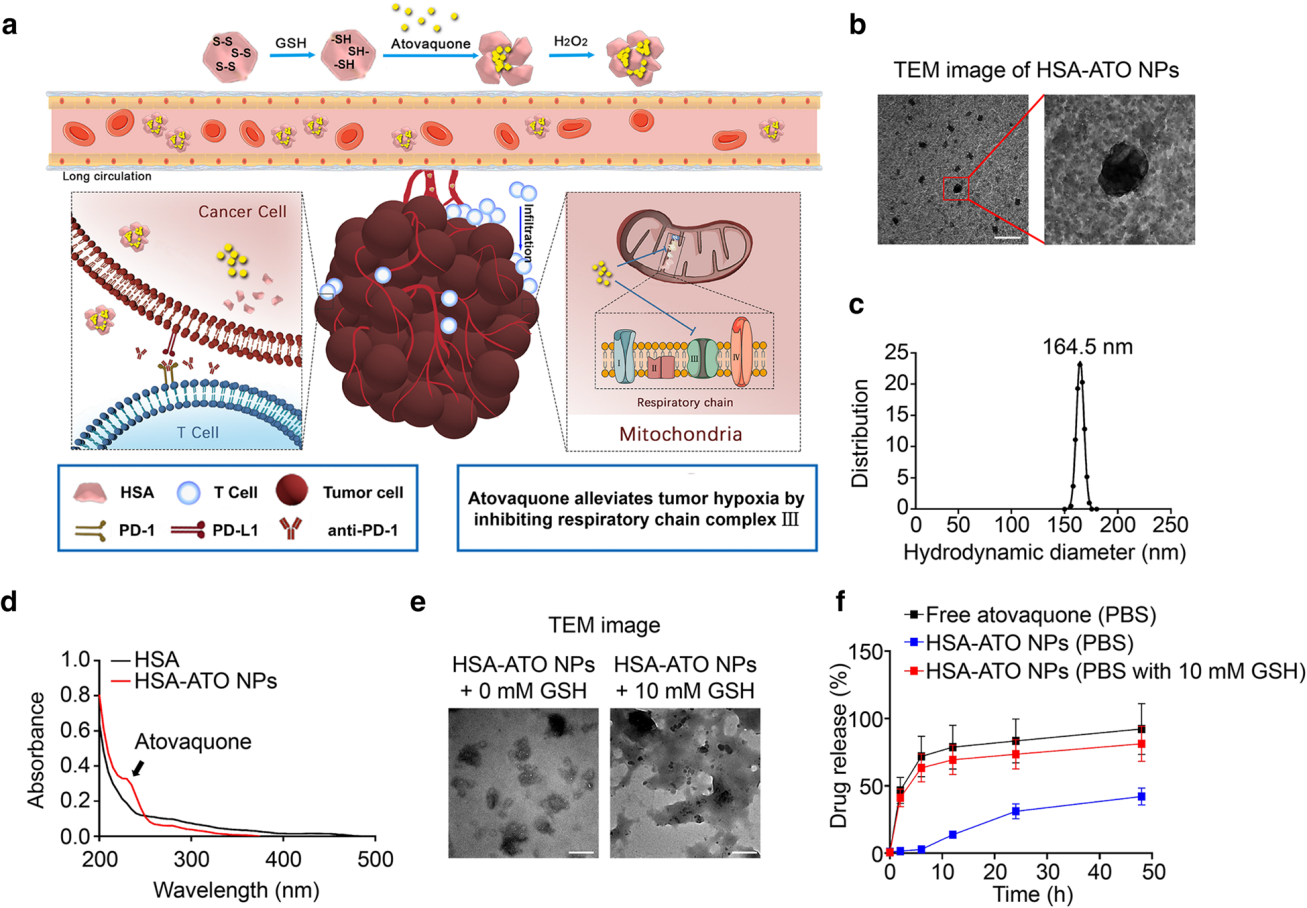
Preparation and characterization of HSA-ATO NPs. **a** Schematic depiction for synthesis and function of HSA-ATO NPs. **b** TEM image of HSA-ATO NPs, presenting an amorphous form. Scale bar: 1 μm. **c** Hydrodynamic distributions of HSA-ATO NPs measured by dynamic light scattering. **d** The full wavelength detection of HSA-ATO NPs and HSA were detected by UV spectrophotometry. **e** TEM image of HSA-ATO NPs response to GSH. Scale bar: 200 nm. **f** In vitro drug release profiles of HSA-ATO NPs in different mediums. Data are presented as mean ± SD

The preparation process of HSA-ATO NPs was shown in Fig. 2a. About 10 mg atovaquone were caught in 100 mg HSA inclusion during the albumin process of denaturation and renaturation, thereby forming nanoparticles composed of an aggregation of multiple HSA proteins. To further investigate the physicochemical properties and loading parameters of HSA-ATO NPs, multiple experiments were shown the characterizations in Table 1. For examples, the zeta potential of HSA-ATO NPs was about − 12.13 mV. The drug loading content of formulations was around 10%, with nearly 90% showing satisfactory entrapment efficiencies. Meanwhile, the morphology of HSA-ATO NPs was represented by TEM (Fig. 2b), and mean size of HSA-ATO NPs was about 164.5 nm (Fig. 2c). To verify whether atovaquone was successfully loaded, we tested various parameters reflecting atovaquone using UV spectrophotometry and HPLC. As shown in Fig. 2d, a new minor peak appeared at 260 nm showing that atovaquone was loaded favorably by albumin.

**Table.1:** Physical characterization of HSA-ATO NPs

HSA-ATO NPs	Mean ± SD (n = 3)
Size (nm)	164.5 ± 10.3
PDI	0.326 ± 0.02
Ζeta potential (mV)	− 12.13 ± 1.05
Drug loading (%)	8.70 ± 1.92
EE (%)	87.2 ± 2.5

*PDI* Polydispersity index, *EE* Encapsulation efficiency

As for the depolymerization character of HSA-ATO NPs, we investigated atovaquone releasing response in PBS buffer (pH 7.4) with or without 10 mmol/L GSH, mimicking the concentration of GSH in tumor cells. The TEM analysis showed that the depolymerization process was triggered when HSA-ATO NPs were incubated at PBS buffer (pH 7.4) with 10 mmol/L GSH for 2 h (Fig. 2e). Meanwhile, after dialysis for 48 h, more than 80% of loaded atovaquone was able to release in PBS buffer with GSH group compare to the free ATO group, while only 30% of the loaded drug was released in PBS buffer without GSH group (Fig. 2f). Taken together, our data indicate that HSA-ATO NPs are successfully constructed as nanoparticles with satisfactory properties.

### HSA-ATO NPs alleviate tumor hypoxia in a PDX model of colon cancer

**Fig. 3:**
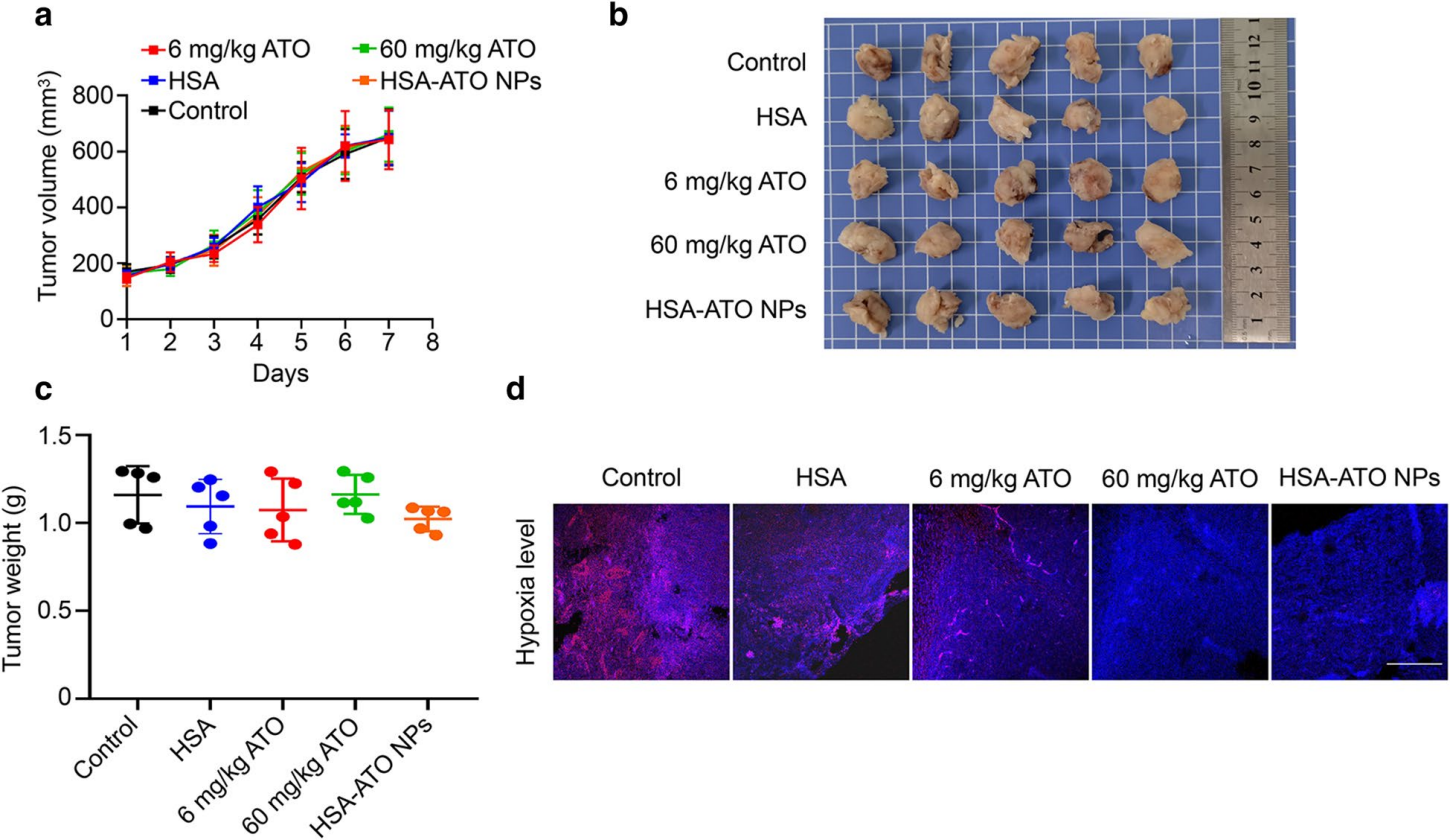
Alleviating tumor hypoxia by HSA-ATO NPs in PDX mouse model. **a** Tumor growth curve of volume according time during the administration (n = 5). Photographs (**b**) and weight (**c**) of tumors collected from mice after 7-day administration (n = 5). **d** APC-Hypoxia probe staining of tumor sections from mice bearing PDX tumors. Scale bar: 500 μm. Data are presented as mean ± SD

To better determine the effect of HSA-ATO NPs on tumor hypoxia in a relatively real tumor microenvironment, we established a PDX mouse model of colon cancer, and administrated these mice with PBS (control), HSA, 6 mg/kg atovaquone (equivalent amounts of atovaquone, corrresponding to those of HSA-ATO NPs) and 60 mg/kg atovaquone (dosage to effectively alleviate tumor hypoxia) and HSA-ATO NPs (atovaquone at a dosage of 6 mg/kg) for 7 days. As expected, HSA-ATO NPs and atovaquone almost did not affect tumor growth (Fig. 3a–c). However, HSA-ATO NPs obviously alleviated tumor hypoxia compared to the control and HSA treatment, which was similar to the atovaquone (60 mg/kg) positive control (Fig. 3d). Besides, our results also showed that 6 mg/kg atovaquone treatment alone (equivalent amounts of atovaquone, corrresponding to those of HSA-ATO NPs) hardly alleviate tumor hypoxia (Fig. 3d). These findings indicate that HSA-ATO NPs is able to effectively alleviate hypoxia environment in solid tumors at a relatively low atovaquone dose, significantly increasing the bioavailability of atovaquone in comparison with previous studies [22].

### HSA-ATO NPs maintain a highly biocompatible profile

**Fig. 4:**
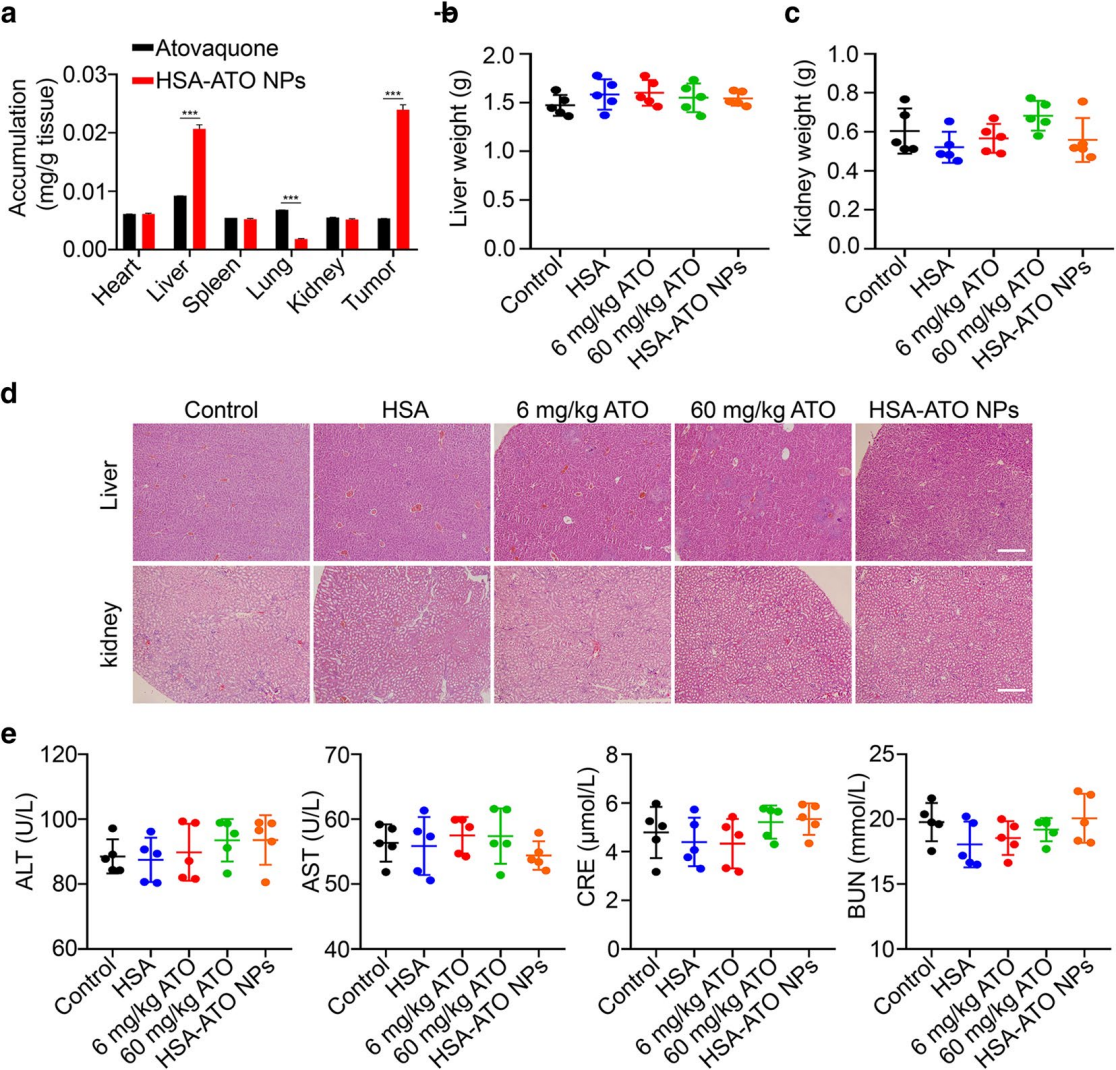
Biosafety evaluation of HSA-ATO NPs. **a** The relative accumulation quality of atovaquone in major organs and tumor sits was analyzed by HPLC. The weight of liver (**b**) and kidney (**c**) of the indicated mice with 7-day administration (n = 5). **d** The representative images of H&E-stained liver and kidney sections in mice with the indicated treatments. Scale bar: 500 μm. **e** The levels of alanine transaminase (ALT), aspartate aminotransferase (AST), serum creatinine (CRE) and blood urea nitrogen (BUN) were analyzed by ELISA assay. Data are presented as mean ± SD. ***, *P* < 0.001

Although all raw materials used in this study are FDA-approved, the biosafety of HSA-ATO NPs is still required further investigation. First of all, we examined drug biodistribution in major organs (including heart, liver, spleen, lung and kidneys) and tumor sites. In general, this kind of nanoparticles with a suitable size (≈ 150 nm) shows a preferable tumor-targeting capacity. As expected, atovaquone was mainly enriched in tumor sites and liver at 6 h after HSA-ATO NPs injection compare to the tumor-bearing mice administrated with equivalent amounts of atovaquone (Fig. 4a). Besides, we also determined the average blood drug concentration-time curves of HSA-ATO NPs and atovaquone, and did not find significant difference in blood concentration-time profiles of atovauone in normal mice after intravenous administration of atovaquone or HSA-ATO NPs (Additional file 1: Figure S2).

Next, 25 mice were treated with PBS (control), HSA, 6 mg/kg atovaquone, 60 mg/kg atovaquone and HSA-ATO NPs for 7 consecutive days. At day 8, major organs and serums were removed and collected to evaluate the biosafety of HSA-ATO NPs. As shown in Fig. 4b, c, HSA-ATO NPs did not affect the liver and kidney weight of mice compared to the mice administered with PBS, HSA, 6 mg/kg atovaquone or 60 mg/kg atovaquone. The results of H&E staining further demonstrated that HSA-ATO NPs did not cause significant systemic toxicity in vivo compared to the control and HSA treatment (Fig. 4d). Besides, there were no notable changes among these five groups in related safety indicators, including the levels of aspartate aminotransferase (AST), alanine transaminase (ALT), serum creatinine (CRE) and blood urea nitrogen (BUN) (Fig. 4e). These data indicate that HSA-ATO NPs maintain a highly biocompatible profile.

### HSA-ATO NPs enhance the efficacy of PD-1 blockade immunotherapy

**Fig. 5:**
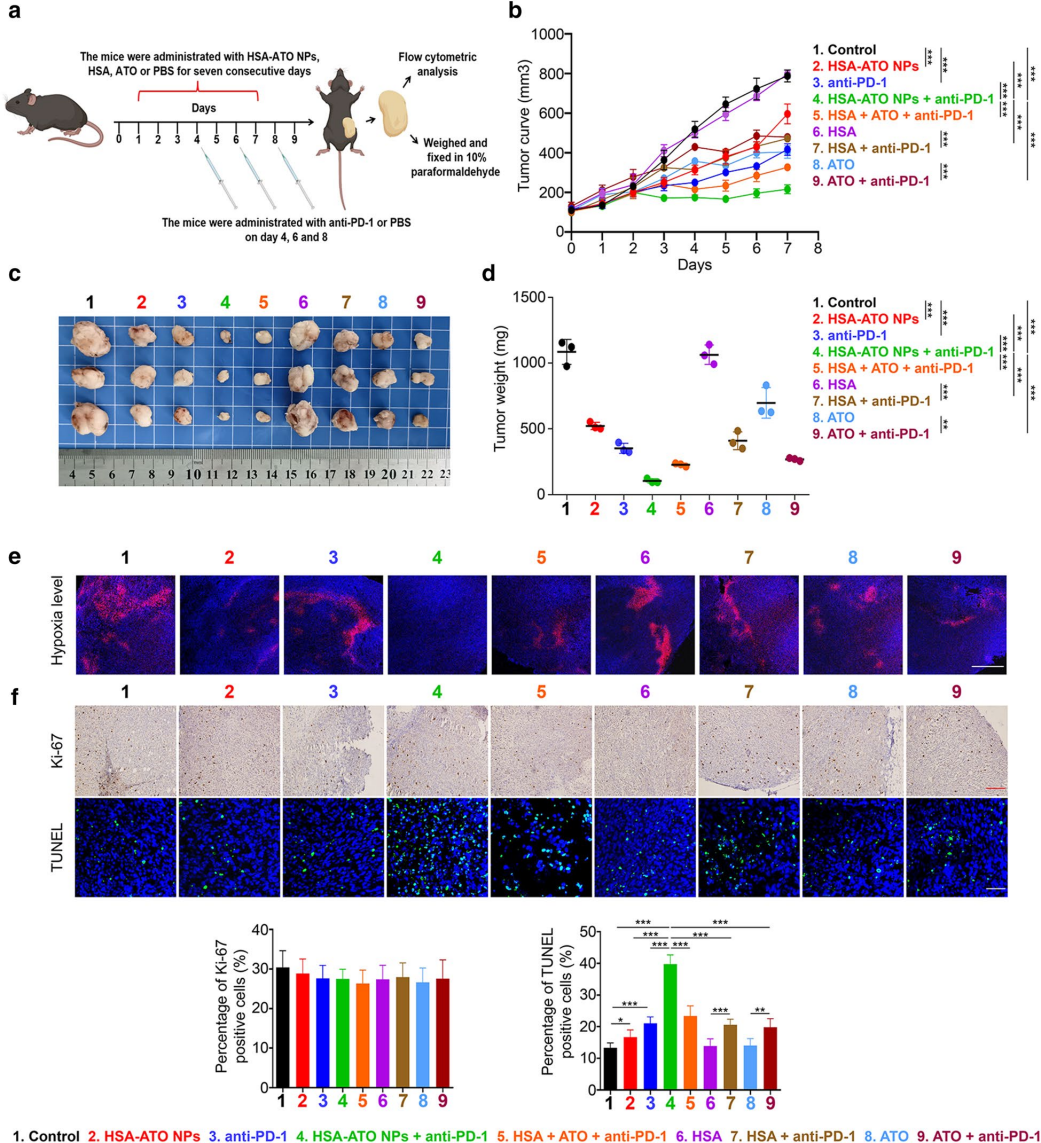
HSA-ATO NPs synergize with anti-PD-L1 therapy. **a** The schematic diagram of MC38 model experimental procedure. **b** Tumor growth curves of volume according time during the administration (n = 6). Photographs (**c**) and weight (**d**) of tumors collected from mice after administration (n = 3). **e** Hypoxia probe staining of tumor sections from mice bearing MC38 tumors. Scale bar: 500 μm. **f** The percentage of Ki-67 and TUNEL positive cells in xenograft tumors was measured by optical (Scale bar: 200 μm) or fluorescence (Scale bar: 50 μm) microscopes. Data are presented as mean ± SD. *, *P* < 0.05; **, *P* < 0.01; ***, *P* < 0.001

It is well-known that blocking PD-1/PD-L1 signaling by PD-1 antibody has been widely used for clinical treatment of cancers [30]. To determine whether HSA-ATO NPs can synergize with anti-PD-1 therapy by alleviating hypoxia environment, we established a homografting colon cancer model by subcutaneously injecting MC38 cells into C57BL6J mice. When the tumors reaching 100 mm^3^, all mice were randomly divided into nine groups (six mice per group): (1) PBS (control); (2) HSA-ATO NPs; (3) anti-PD-1; (4) HSA-ATO NPs + anti-PD-1; (5) HSA + ATO + anti-PD-1; (6) HSA; (7) HSA + anti-PD-1; (8) ATO; (9) ATO + anti-PD-1. The amount of atovaquone in traditional intragastric administration was equal to the HSA-ATO NPs (6 mg/kg). The schematic diagram of MC38 model experimental procedures is illustrated in Fig. 5a. As shown in Fig. 5b–d, combined treatment of HSA-ATO NPs and anti-PD-1 synergistically inhibited tumor growth compared to anti-PD-1 or HSA-ATO NPs treatment alone, also better than other single or combined treatment. As expected, we found that hypoxia was effectively alleviated in the groups of HSA-ATO NPs and HSA-ATO NPs + anti-PD-1, while other groups still remain varying degrees of hypoxia (Fig. 5e).

Notably, despite the Ki-67 levels in tumor tissues did not show any significant difference among combined treatment, anti-PD-1 or HSA-ATO NPs treatment alone, the number of TUNEL-positive cells in the tumors with combined treatment of ATO NPs and anti-PD-1 was much higher than that in the tumors with anti-PD-1 or HSA-ATO NPs treatment alone (Fig. 5f). Taken together, our data indicate that HSA-ATO NPs effectively sensitize anti-PD-1 therapy, and further demonstrate that HSA-ATO NPs greatly improve efficacy of PD-1 blockade immunotherapy.

### HSA-ATO NPs alleviate immunosuppressive tumor immune microenvironment (TIME)

**Fig. 6:**
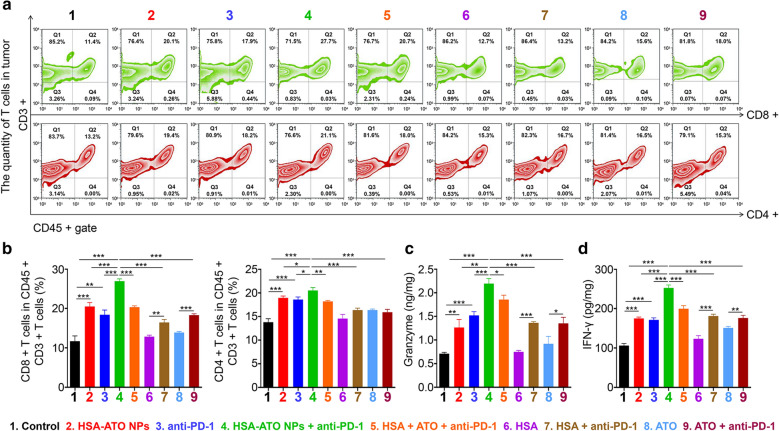
Relief of immunosuppressive tumor immune microenvironment by HSA-ATO NPs. **a** Flow cytometry analysis of CD4+/CD8+ cells in tumors from mice with the indicated treatments. The quantity of immune cells (**b**) and the content of Granzyme (**c**) and IFN-γ (**d**) in xenograft tumors from mice with the indicated treatments were analyzed by immunofluorescence and ELISA assays. Data are presented as mean ± SD. *, *P* < 0.05; **, *P* < 0.01; ***, *P* < 0.001

To explore how HSA-ATO NPs improve PD-1 blockade immunotherapy, we next investigated the infiltration of cytotoxic T lymphocyte (CTL) in xenograft tumors. CD8+ cytotoxic T cells, as the most common CTLs, are a key effector in tumor cell eradication. Flow cytometry analysis showed that CD8+ T cell infiltration in the tumors with combined treatment of HSA-ATO NPs and anti-PD-1 was the most obvious compared to other treatments (Fig. 6a, b). Similarly, the quantities of CD4+ T cells also showed the same trend (Fig. 6a, b). Besides, the granzyme and IFN-γ, commonly associated with the immune cell activities, were clearly elevated by combined treatment of HSA-ATO NPs and anti-PD-1 compared to other treatments (Fig. 6c). These observations indicate that HSA-ATO NPs enhance the efficacy of anti-PD-1 therapy by effectively alleviating immunosuppressive TIME.

## Discussion

In this study, we link hypoxic tumor microenvironment to the resistance of immunotherapy, further highlighting the importance of tumor hypoxic microenvironment in cancer therapy. Our results reveal that hypoxia is an important barrier for T cell infiltration, thereby limiting the efficacy of immunotherapy. Improving tumor microenvironment by inhibition of tumor cell oxidative metabolism will significantly increase sensitivity to immunotherapy, unleashing antitumor immune response to promote cancer regression [20, 31, 32]. This was strongly supported by our data that both quantity and activity of the immune cells were increased after improving tumor microenvironment [33, 34]. Taken together, the situation of tumor environment directly determines the efficacy of cancer immunotherapy. Thus, tumor microenvironment has to be considered for cancer therapy, especially hypoxia environment [35, 36].

In general, two strategies “direct delivery of oxygen to tumor” and “reduce tumor oxygen consumption” are often designed to alleviate tumor hypoxia. However, the latter method is more advantageous for achieving with the drugs. There is evidence showing that normalizing tumor hypoxia by existing drugs is experimentally feasible for clinical treatment of cancers [22]. In this study, our data strongly indicated that atovaquone, an FDA-approved drug for malaria treatment, significantly alleviated the harsh hypoxic tumor microenvironment. Although other drugs can also achieve similar effects by inhibiting tumor oxygen consumption, atovaquone exhibits the most powerful effect on lowering tumor oxygen consumption. More importantly, it does not exert toxic effects for major organs at an adequate dose to alleviate hypoxia [20]. However, the extremely low water-solubility of atovaquone leads to its poor bioavailability, thereby greatly limiting its clinical utility. It is very necessary to improve its bioavailability in order to meet clinical needs. Thus, designing a proper delivery system will be an effective strategy to address the above mentioned dilemma.

Albumin has good biocompatibility and biodegradability, thus serving as a versatile carrier for drug delivery [37]. One of the best known examples is albumin-bound paclitaxel (nab-PTX), which has been used in clinic for years [38]. Paclitaxel is enwrapped into albumin to form stable nanoparticles with a suitable size in order to enhance the bioavailability of PTX [38]. Considering that atovaquone is as hydrophobic as PTX, this kind of nanoparticle will provide an effective strategy to solve delivery problem of atovaquone [39]. Not surprisingly, our data showed that the albumin delivery system was able to load atovaquone, which formed nanoparticles named HSA-ATO NPs. Compared to traditional oral drug administration, the dose of HSA-ATO NPs to normalize tumor hypoxia was drastically reduced by as much as 10-fold. Under the premise of the same curative effect, this kind of nano-drugs greatly reduces the dose of atovaquone for clinical treatment. Moreover, due to the EPR effect and specific albumin receptors in malignant tumors, HSA-ATO NPs possessed the excellent targeting efficiency to tumor [24, 27, 40]. This also verified that albumin delivery system increased the bioavailability of the loaded drugs and maintained a highly biocompatible profile.

It is the fact that clinical tumors are highly heterogeneous in terms of tumor microenvironment containing endothelial cells, pericytes, immune cells, fibroblasts and extracellular matrix (ECM) [41–43]. The xenograft tumor model derived from a single cell line can not fully reflect complete tumor microenvironment, while PDX model will effectively mimic the native tumor microenvironment [44, 45]. In this study, our data demonstrated that the HSA-ATO NPs effectively alleviated tumor hypoxia in PDX mouse model, indicating that this kind of nano-drug has a great potential for the treatment of cancer patients. Importantly, all raw materials used in this study are FDA-approved, thus HSA-ATO NPs are all considered as safe and reliable in clinic. Based on this system, a number of improvements also could be made. One of them is that make the checkpoint antibodies directly conjugate to albumin by unique chemical modifications. As a consequence, this will form a more complex nano-drugs to solve a variety of problems, such as the drugs with low bioavailability or poor tumor targeting. In the near future, we may carry out other combined treatment regimens by this safe and targeting delivery system. Thus, we are going to persist exploiting this system to investigate more issues.

## Conclusions

In summary, we develop a novel nano-drug by using HSA to load atovaquone, and demonstrate that this nano-drug enhances the efficacy of PD-1 blockade immunotherapy by alleviating tumor hypoxia microenvironment, thereby providing a potential strategy for cancer therapy.

## Supplementary Information

**Additional file 1: Figure S1.** In vitro toxicity evaluation of atovaquone. **Figure S2.** The average blooddrug concentration-time curves of HSA-ATO NPs and atovaquone.

## Data Availability

The datasets generated and analyzed during the current study are available from the corresponding author on reasonable request.

## Abbreviations

HSA: Human serum albumin
PD-1: Programmed death-1
PD-L1: Programmed death ligand 1
FDA: Food and Drug Administration
TEM: Transmission electron microscope
NPs: Nanoparticles
ELISA: Enzyme-linked immunosorbent assay
CD8: Cluster of differentiation 8
CD4: Cluster of differentiation 4
CTLA4: Cytotoxic T lymphocyte-associated protein 4
EPR: Enhanced permeability and retention
gp60: Glycoprotein 60
DMSO: Dimethylsulfoxide
EE: Entrapment efficiency
HPLC: High performance liquid chromatography
DMEM: Dulbecco’s Modified Eagle Medium
FBS: Fetal bovine serum
GSH: Glutathione
OCR: Oxygen consumption rates
PDX: Patient-derived xenograft
H&E: Hematoxylin and eosin
AST: Aspartate aminotransferase
ALT: Alanine transaminase
BUN: Blood urea nitrogen
CRE: Creatinine
OXPHOS: Oxidative phosphorylation
IFN-γ: Interferon-γ
TIME: Tumor immune microenvironment
CTL: Cytotoxic T lymphocyte
nab-PTX: Albumin-bound paclitaxel
PTX: Paclitaxel
ECM: Extracellular matrix
