# Supplementary material for: Atovaquone-HSA nano-drugs enhance the efficacy of PD-1 blockade immunotherapy by alleviating hypoxic tumor microenvironment
Source: J Nanobiotechnology. 2021 Oct 2;19:302. doi: 10.1186/s12951-021-01034-9 (PMC8487475; doi:10.1186/s12951-021-01034-9)
Supplement: Supplementary file 1 — Additional file 1: Figure S1. In vitro toxicity evaluation of atovaquone. Figure S2. The average blooddrug concentration-time curves of HSA-ATO NPs and atovaquone. [file 12951_2021_1034_MOESM1_ESM.docx]

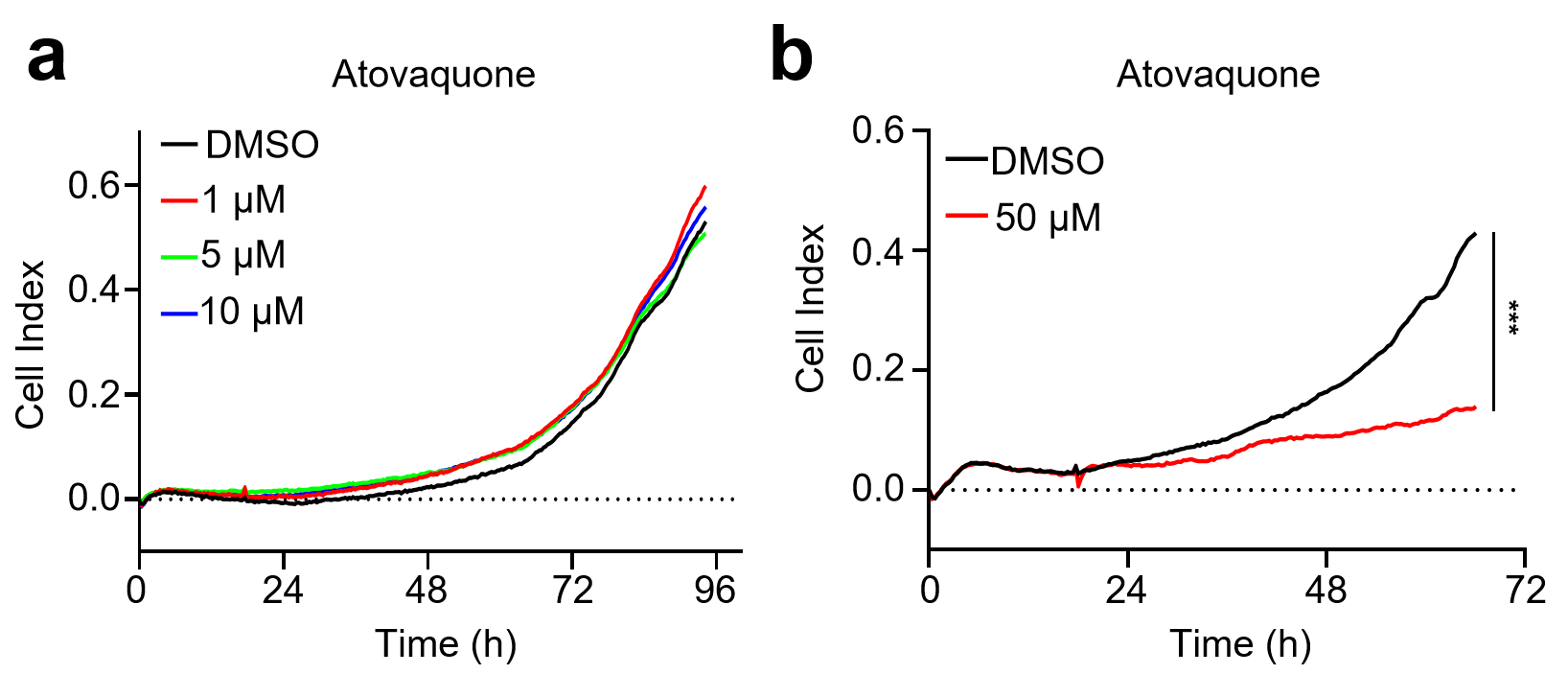


**Figure S1. *In vitro* toxicity evaluation of atovaquone.** The viability of MC38 cells (3000/well) treated with different concentrations (**a**, 1, 5 and 10 μM; **b**, 50 μM) of atovaquone was examined by a Real-Time Cell Analysing (RTCA, Agilent Technologies, USA) during the 3-4 days. Data are presented as mean ± SD. ***, *P* <0.001.


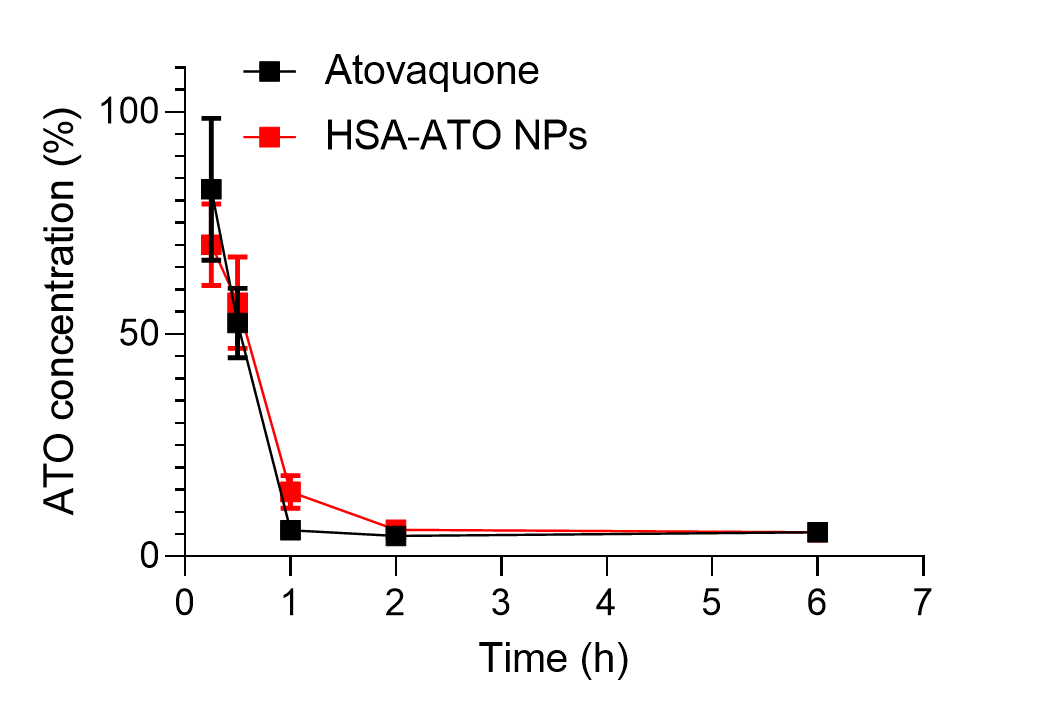


**Figure S2. The average blood drug concentration-time curves of HSA-ATO NPs and atovaquone.** Blood concentration-time profiles of atovauone in normal mice after intravenous administration of atovaquone or HSA-ATO NPs (0.25, 0.5, 1, 2 and 6 h, n = 3).
